# Fedorov algorithm–optimized chemometric spectrophotometry for cefepime–tazobactam microanalysis in plasma and pharmaceuticals with integrated MA and NQS sustainability assessment

**DOI:** 10.1038/s41598-026-55675-7

**Published:** 2026-06-05

**Authors:** Asmaa G. Gad, Khadiga M. Kelani, Amr M. Mahmoud, Reham M. Arafa, Ahmed Emad F. Abbas

**Affiliations:** 1 Postgraduate Program in Pharmaceutical Analytical Chemistry Department, Faculty of Pharmacy, Cairo University, El-Kasr El-Aini Street, Cairo, 11562 Egypt; 2 Pharmaceutical Analytical Chemistry Department, Faculty of Pharmacy, Modern University for Technology and Information, Cairo, Egypt; 3 Pharmaceutical Analytical Chemistry Department, Faculty of Pharmacy, Cairo University, El-Kasr El-Aini Street, Cairo, 11562 Egypt; 4 Analytical Chemistry Department, Faculty of Pharmacy, October 6 University, 6 October City, Giza, 12585 Egypt

**Keywords:** Cefepime–tazobactam, Chemometric spectrophotometry, Fedorov optimal design, Multivariate curve resolution (MCR-ALS), Firefly algorithm, Sustainability assessment, Green analytical chemistry, Chemistry, Environmental sciences

## Abstract

A green, sustainability-oriented chemometric-assisted UV spectrophotometric platform was developed for the simultaneous determination of Cefepime (CFPM) and Tazobactam (TAZO) in pharmaceutical formulations and human plasma. Water served as the sole diluent, eliminating hazardous organic solvents and substantially reducing the environmental impact of the analytical procedure. Calibration and validation sets were efficiently constructed using the Fedorov exchange algorithm within Brereton’s multilevel design framework, yielding 25 calibration and 13 validation mixtures and reducing experimental workload by approximately 70% compared with conventional designs. Three complementary chemometric models were evaluated: Principal Component Regression (PCR), Firefly Algorithm–assisted Partial Least Squares (FA-PLS), and Multivariate Curve Resolution–Alternating Least Squares (MCR-ALS). Among them, MCR-ALS demonstrated superior predictive performance, with correlation coefficients exceeding 0.9998 for both analytes, minimal systematic bias confirmed by Elliptical Joint Confidence Region analysis, and high robustness across pharmaceutical and plasma matrices. Limits of detection were 0.0487 and 0.0396 µg mL^–1^, while limits of quantification were 0.1476 and 0.1200 µg mL^–1^ for CFPM and TAZO, respectively. The method was validated in accordance with ICH guidelines and successfully applied to CEFE-MAX™ powder for injection and fortified human plasma. Matrix effect evaluation showed negligible signal suppression (− 2.35% to − 1.27%), indicating strong matrix tolerance and calibration transferability. Comparative benchmarking against reported HPLC–UV, LC–MS/MS, capillary zone electrophoresis, and UV spectrophotometric methods demonstrated that the proposed approach achieves an optimal balance of sensitivity, simplicity, and environmental sustainability. Sustainability assessment using the Multi-Color Assessment (MA), carbon footprint analysis, and the Need–Quality–Sustainability (NQS) index yielded a Whiteness Score of 83.6%, an NQS score of 90, and a low carbon footprint of 0.032 kg CO₂-eq per analysis. Overall, the developed platform provides a rapid, cost-effective, and environmentally responsible alternative for routine quality control and preliminary therapeutic monitoring of the CFPM–TAZO combination.

**Supplementary Information:**

The online version contains supplementary material available at 10.1038/s41598-026-55675-7.

## Introduction

Pharmaceutical analysis has undergone notable evolution in recent years as the scientific community increasingly emphasizes analytical methodologies that combine accuracy, efficiency, and environmental responsibility. Conventional quality control of multicomponent pharmaceutical formulations frequently relies on chromatographic separation techniques. Although these methods provide excellent sensitivity and selectivity, they are often associated with sophisticated instrumentation, considerable solvent consumption, and relatively long analysis times. In the context of modern sustainable laboratory practices, there is growing interest in developing alternative analytical approaches that deliver reliable performance while minimizing chemical use, operational complexity, and analytical waste. Among available analytical tools, UV–visible spectrophotometry remains one of the most accessible and environmentally favorable techniques due to its simplicity, rapid data acquisition, and low operational cost. Furthermore, spectrophotometric measurements can typically be performed in aqueous media, thereby reducing the analytical workflow’s environmental footprint^1–3^. Nevertheless, the direct spectrophotometric determination of multicomponent pharmaceutical systems is frequently challenged by severe spectral overlaps among coexisting analytes. Under such circumstances, traditional univariate calibration methods are inadequate and require advanced computational tools to extract quantitative information from highly overlapping spectral signals^4–6^.

In parallel with these analytical challenges, the widespread presence of substandard and falsified medicines continues to pose a significant concern for global healthcare systems^7^. Quality deficiencies may arise from inadequate manufacturing practices, improper storage conditions, degradation during distribution, or intentional falsification of pharmaceutical products^8^. Such irregularities may lead to deviations from the declared active pharmaceutical ingredient (API) content or the presence of inappropriate formulations, potentially compromising therapeutic outcomes and patient safety. Consequently, the development of reliable analytical methodologies capable of verifying pharmaceutical composition remains essential for regulatory quality control and routine monitoring throughout the drug supply chain^9^.

Cefepime (CFPM), a fourth-generation cephalosporin, exerts bactericidal activity through inhibition of bacterial cell wall biosynthesis. It demonstrates broad-spectrum coverage against Gram-positive and Gram-negative pathogens and is frequently indicated in severe systemic infections (Fig. S1). Tazobactam (TAZO), a β-lactamase inhibitor, is co-administered to protect CFPM from enzymatic hydrolysis by β-lactamase-producing microorganisms. The CFPM–TAZO combination, therefore, represents a clinically valuable synergistic system designed to overcome bacterial resistance mechanisms, Fig. S1.

Given the therapeutic significance of this combination, reliable and sensitive analytical methodologies are essential for quality control, regulatory compliance, and therapeutic monitoring. Reported procedures for the simultaneous determination of CFPM and TAZO have predominantly relied on chromatographic platforms including UHPLC^10^, HPTLC^11^, capillary zone electrophoresis^12^, MS/MS^13^, RP-HPLC^14^, and conventional HPLC^15–17^ in addition to few spectrophotometric methods^18,19^. Although these techniques provide high selectivity and sensitivity, they are frequently associated with extensive consumption of hazardous organic solvents, elevated operational costs, prolonged analysis time, and substantial environmental burden. From a sustainability perspective, routine reliance on solvent-intensive chromatographic systems is increasingly inconsistent with modern green analytical chemistry (GAC) principles.

Chemometric-assisted multivariate calibration provides an effective strategy to overcome spectral overlap limitations in spectrophotometric analysis. By exploiting latent information embedded within spectral datasets, chemometric models enable simultaneous quantification of multiple components without the need for physical separation. Regression-based techniques such as Principal Component Regression (PCR) and Partial Least Squares (PLS) utilize covariance relationships between spectral variables and analyte concentrations to construct predictive calibration models. In contrast, Multivariate Curve Resolution–Alternating Least Squares (MCR-ALS) employs bilinear matrix decomposition to recover both concentration profiles and pure spectral signatures of the analytes under appropriate constraints, offering enhanced interpretability and flexibility^20,21^. In recent years, the integration of metaheuristic optimization algorithms, such as the Firefly Algorithm (FA), with PLS modeling has been explored to improve variable selection and enhance predictive accuracy. Despite these advances, the predictive reliability of chemometric calibration models strongly depends on the design and distribution of calibration and validation samples^22,23^.

Conventional experimental layouts based on random or full-factorial mixture generation may introduce redundant data points, increase experimental workload, and unnecessarily inflate chemical consumption. Optimal experimental design theory provides a rational solution to this problem by identifying the most informative experimental subsets. The Fedorov exchange algorithm represents a powerful optimization tool in this context, as it iteratively selects experimental points that maximize statistical information while minimizing variance inflation according to predefined optimality criteria. Although widely applied in statistical modeling, its application in chemometric-assisted spectrophotometric analysis of pharmaceutical combinations remains relatively limited^24,25^.

Alongside analytical performance, sustainability considerations have become increasingly important in modern analytical chemistry. Contemporary evaluation frameworks extend beyond classical validation parameters to incorporate environmental impact, resource utilization, and operational practicality. Multidimensional sustainability assessment tools such as the Multi-Color Assessment (MA) metric allow simultaneous evaluation of analytical performance, ecological impact, and methodological practicality, yielding an integrated “whiteness” score. Similarly, the Need–Quality–Sustainability (NQS) index integrates methodological necessity, analytical robustness, and environmental responsibility into a single quantitative indicator. When complemented by carbon footprint estimation, these metrics provide a comprehensive framework for benchmarking analytical methods within the context of sustainable laboratory practices.

In light of these considerations, the present study proposes a sustainable chemometric-assisted UV spectrophotometric platform for the simultaneous microdetermination of CFPM and TAZO in pharmaceutical formulations and human plasma. The analytical workflow employs water as the sole diluent, thereby eliminating hazardous organic solvents and reducing environmental impact. Calibration and validation matrices were constructed using the Fedorov exchange algorithm to achieve an information-optimal experimental design with significantly reduced experimental runs. Three chemometric paradigms—PCR, Firefly-optimized PLS, and MCR-ALS—were comparatively evaluated in terms of predictive performance and statistical robustness.

To the best of our knowledge, this is the first study to integrate a Fedorov exchange algorithm–based experimental design, advanced chemometric modeling, application to human plasma with matrix effect evaluation, and comprehensive sustainability benchmarking for the simultaneous determination of CFPM and TAZO. In addition, the sustainability profile of the proposed methodology was thoroughly assessed using the Multi-Color Assessment (MA) framework, carbon footprint analysis, and the Need–Quality–Sustainability (NQS) index, and benchmarked against representative reported methods, including HPLC–UV, LC–MS/MS, capillary zone electrophoresis, and conventional UV spectrophotometry. This integrated analytical framework combines optimal experimental design, multivariate modeling, and quantitative sustainability evaluation to provide a robust, environmentally responsible, and practically applicable alternative for modern pharmaceutical analysis.

## Experimental

### **Instrumentation and software**

Spectrophotometric analyses were carried out using a Shimadzu UV-1800 double-beam UV–Visible spectrophotometer (Shimadzu Corporation, Kyoto, Japan; www.shimadzu.com), equipped with matched 1.0 cm quartz cuvettes (Hellma Analytics, Müllheim, Germany; www.hellma.com). The instrument was optimized for high-precision measurements under the following conditions: a spectral bandwidth of 1.0 nm, a sampling interval of 0.5 nm, and a medium scanning speed to achieve an optimal balance between resolution and signal-to-noise ratio. All measurements were performed at a controlled room temperature of 25 ± 0.5 °C. Baseline correction and instrument operation were managed using UV-Probe software (version 2.62, Shimadzu Corporation, Kyoto, Japan).

All weighing procedures were performed using an analytical balance (Shimadzu AUX-220, Shimadzu Corporation, Kyoto, Japan; readability: 0.1 mg, linearity: ±0.2 mg), with internal calibration verified daily to ensure measurement accuracy. Sample dissolution was achieved using a Julabo USC300TH ultrasonic bath (Julabo GmbH, Seelbach, Germany; www.julabo.com) operating at 40 kHz and equipped with precise digital temperature control (± 0.1 °C), ensuring complete dissolution of samples while preventing thermal degradation of temperature-sensitive analytes.

Chemometric analyses were conducted using MATLAB R2023a (v9.14.0, MathWorks Inc., Natick, MA, USA; www.mathworks.com) integrated with PLS Toolbox v9.1 (Eigenvector Research Inc., Manson, WA, USA; www.eigenvector.com) and MCR-ALS Toolbox v2.1 (Multivariate Curve Resolution Toolbox, Universität de Barcelona, Spain; www.mcrals.info). Optimal experimental design and validation set selection were performed using the Fedorov algorithm implemented through MATLAB’s Statistics and Machine Learning Toolbox (MathWorks Inc., USA) based on D-optimal criteria. Metaheuristic learning algorithms) FA-PLS, were executed via MATLAB’s Global Optimization Toolbox (MathWorks Inc., USA).

### Materials and chemicals

#### Reference standards

Certified reference standards of CFPM and TAZO (Fig. S1) were kindly supplied by the National Organization for Drug Control and Research (Cairo, Egypt). Purity assessment was performed according to the official pharmacopeial method for CFPM and a validated reported procedure for TAZO. The certified purity values were 100.14% ± 0.90 for CFPM and 99.6% ± 0.80 for TAZO.

Human plasma was purchased from an internationally accredited holding company (VACSERA, Cairo, Egypt).

#### Pharmaceutical formulations

CEFE-MAX™ powder for injection was procured from the commercial market (batch No. IABG8005A). The labeled claim corresponds to 1.00 g CFPM and 125.00 mg TAZO per vial.

#### Solvents and reagents

Ultra-pure water (resistivity 18.2 MΩ·cm at 25 °C; total organic carbon < 2 ppb) was obtained from a Milli-Q IQ 7000 purification system (Millipore, USA). Before use, water was passed through 0.22 μm membrane filters to eliminate particulate matter and ensure spectrophotometric transparency. No organic solvents were employed throughout the analytical procedure.

### Standard solution preparation

Primary stock solutions (1.00 mg mL^–1^) of CFPM and TAZO were prepared separately by accurately weighing 50.0 mg of each standard into 50-mL volumetric flasks and dissolving in ultra-pure water. Dissolution was facilitated by ultrasonic agitation for 20 min at ambient temperature.

Stock solutions were stored at 2–8 °C in amber glass containers. Stability was verified over a 21-day period under refrigerated conditions.

Working solutions were freshly prepared by serial dilution of the stock solutions using ultra-pure water. An intermediate solution (100.0 µg mL^–1^) was employed to minimize cumulative volumetric error. Calibration concentrations were adjusted to cover the analytical ranges of 1.00–17.00 µg mL^–1^ for CFPM and 1.00–9.00 µg mL^–1^ for TAZO. All solutions were prepared using class A volumetric glassware.

### Spectroscopic data characteristics and working range assessment

**Fig. 1:**
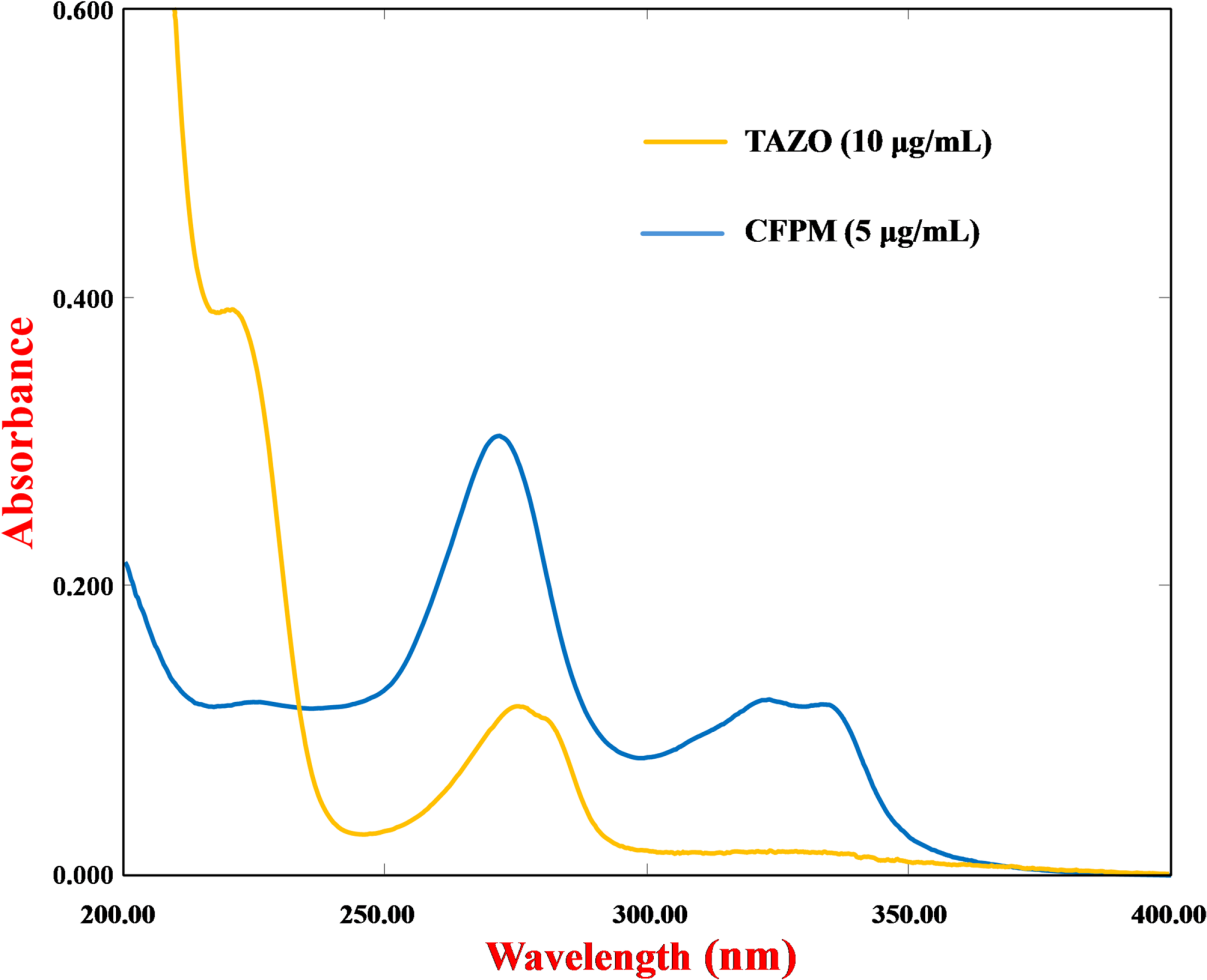
Zero-order absorption spectra of TAZO and CFPM.

The absorption spectra of CFPM and TAZO were recorded over the 200–450 nm wavelength interval to characterize their intrinsic spectral behavior and define the analytical measurement window (Fig. 1). Spectral scans were obtained using aqueous standard solutions containing 5.0 µg mL^–1^ CFPM and 10.0 µg mL^–1^ TAZO. Ultra-pure water served as the reference blank.

Initial spectral acquisition was performed at 1.0 nm resolution across the full wavelength domain to ensure comprehensive capture of chromophoric transitions. For subsequent multivariate processing, the operative spectral region was restricted to 210–350 nm, yielding 141 discrete wavelength variables. The analytical working concentration domains were established through preparation of systematic dilution series spanning 1.00–17.00 µg mL^–1^ for CFPM and 1.00–9.00 µg mL^–1^ for TAZO. These ranges were adopted to encompass the formulation ratio while maintaining spectrophotometric proportionality within the instrument’s linear dynamic response. All spectral matrices used for chemometric model construction were derived from this optimized wavelength interval and concentration domain.

### Experimental design and optimization strategy

A dual-phase design architecture was therefore adopted, integrating structured calibration matrix construction with algorithm-optimized validation subset selection. Calibration mixtures were generated according to Brereton multilevel design principles to ensure uniform population of the bidimensional concentration domain. The calibration set comprised 25 binary mixtures distributed across the analytical working ranges of 1.00–17.00 µg mL^–1^ for CFPM and 1.00–9.00 µg mL^–1^ for TAZO. Concentration limits were defined in accordance with the validated spectrophotometric linearity interval and the formulation ratio of the investigated pharmaceutical product. Independent validation samples were selected using the Fedorov exchange algorithm to achieve an information-optimal external test set. The final validation set consisted of 13 mixtures exhibiting minimal leverage redundancy and homogeneous spatial distribution. All designed mixtures were prepared freshly into 25-mL volumetric flasks, with volume completion using ultra-pure water prior to spectral acquisition.

### Multivariate calibration modeling and algorithmic configuration

To resolve spectral collinearity within the binary CFPM–TAZO system, three complementary chemometric frameworks representing regression-, learning-, and resolution-based paradigms were constructed. Model development was performed using the Fedorov-structured calibration matrix (*n* = 25), whereas predictive generalization was examined using the independent validation subset (*n* = 13).

PCR modeling was implemented following preprocessing of the spectral data matrix through mean-centering and variance scaling to normalize variable magnitude and enhance numerical conditioning. Dimensionality reduction was achieved via singular value decomposition, generating orthogonal latent variables describing the principal variance structure of the dataset. Selection of the optimal factor dimensionality was guided by cross-validation using a minimum prediction residual criterion. Final calibration functions were derived by regressing analyte concentration vectors against the retained principal component score matrix.

Learning-assisted wavelength selection was conducted using the FA coupled with PLS regression. Within this metaheuristic framework, each firefly encoded a candidate spectral variable subset. Attractiveness between fireflies was modulated according to objective fitness values derived from cross-validated calibration residuals. Iterative population updating was governed by light absorption coefficients and stochastic perturbation parameters to balance exploitation and exploratory search behavior. Converged wavelength subsets were subsequently processed through PLS latent variable modeling, with factor dimensionality determined through internal cross-validation.

Resolution of overlapping spectral contributions was performed using the MCR-ALS bilinear decomposition algorithm. Initial estimations of pure component spectra and concentration profiles were generated through Evolving Factor Analysis to define system rank and extract preliminary loading vectors. Alternating least squares optimization was then executed under chemically relevant constraints. Non-negativity was imposed on both concentration and spectral matrices, while closure constraints preserved mixture compositional balance. Spectral selectivity constraints were applied to maintain realistic chromophoric band structure, and concentration boundaries were restricted within the experimental design domain. Iterative refinement proceeded until convergence was achieved according to residual stabilization criteria.

Prior to model construction, spectral datasets were subjected to standardized preprocessing routines including baseline offset correction, noise smoothing, and autoscaling to ensure inter-model comparability and computational stability.

#### Chemometric model selection criteria

Selection of the multivariate calibration frameworks was guided by the need to interrogate the CFPM–TAZO system through complementary algorithmic perspectives encompassing regression, learning-assisted optimization, and bilinear resolution.

PCR was adopted as a foundational latent-variable regression technique to establish a transparent linear calibration benchmark. Owing to its orthogonal factor extraction and variance-compression structure, PCR enables systematic handling of spectral collinearity while preserving interpretability of the calibration space.

FA-PLS was incorporated to represent a learning-optimized regression paradigm. In this hybrid configuration, metaheuristic wavelength selection is embedded within the PLS framework to refine variable subsets prior to latent variable modeling. The FA, operating through attractiveness-driven swarm intelligence mechanics, facilitates localization of spectrally informative regions while suppressing redundant or noise-dominated variables.

MCR-ALS was selected as a resolution-oriented approach capable of decomposing bilinear spectral datasets into pure component signatures and corresponding concentration distributions without reliance on explicit calibration regressors. Under appropriate physicochemical constraints, MCR-ALS enables recovery of chemically meaningful profiles and demonstrates resilience toward structured spectral interferences.

The combined deployment of PCR, FA-PLS, and MCR-ALS provides a tiered analytical framework spanning classical factor regression, machine learning–assisted optimization, and constrained bilinear resolution, thereby enabling comprehensive interrogation of severely overlapping spectroscopic systems.

### Validation protocol and statistical evaluation framework

Analytical validation of the proposed spectrophotometric–chemometric platform was conducted in accordance with the International Council for Harmonisation (ICH) Q2(R1) guideline for analytical procedure validation, with procedural alignment to the updated Q2(R2) and Q14 guidance documents addressing analytical development lifecycle management. Validation design encompassed assessment of acuraccy, precision, sensitivity, and method resilience using calibration and external validation datasets^26^. Model predictive capability was quantified using multivariate calibration error diagnostics calculated from reference (y_i_) and predicted (ŷ_i_) concentration vectors across n samples. Evaluated parameters included the root mean square error of calibration (RMSEC), root mean square error of prediction (RMSEP), relative RMSEP (RRMSEP), bias, bias-corrected RMSEP (BCRMSEP), and standard error of calibration (SEC). Mathematical expressions were applied as follows:

$$RMSE=\sqrt {\frac{{\mathop \sum \nolimits_{{i=1}}^{n} {{\left( {yi~ - ~\hat {y}i} \right)}^2}}}{n}}$$

$$Bias=\frac{{\mathop \sum \nolimits_{{i=1}}^{n} \left( {yi~ - ~\hat {y}i} \right)}}{n}$$

$$SEC=\sqrt {\frac{{\mathop \sum \nolimits_{{i=1}}^{n} {{\left( {yi~ - ~\hat {y}i~ - bias} \right)}^2}}}{{n - 1}}}$$

$$RRMSEP\% =\frac{{\frac{1}{n}\sqrt {\mathop \sum \nolimits_{{i=1}}^{n} {{\left( {yi~ - ~\hat {y}i~} \right)}^2}} }}{{\bar {y}i}} \times 100$$

where $\bar{y}$ represents the mean reference concentration.

Limits of detection (LOD) and quantification (LOQ) were estimated using the net analyte signal (NAS) approach to account for spectral overlap and multivariate background contributions. Method Accuracy was evaluated through recovery analysis at three concentration levels spanning the analytical domain. Test solutions were prepared at 5.0, 10.0, and 15.0 µg mL^–1^ for CFPM and 3.0, 5.0, and 7.0 µg mL^–1^ for TAZO. Each level was analyzed in sextuplicate, and percentage recovery values were computed relative to nominal concentrations. Precision was investigated at two hierarchical levels: Repeatability (intra-day precision): Six replicate determinations performed within a single analytical session and intermediate precision (inter-day precision): Replicate analyses conducted over three consecutive days under identical operational conditions. Method robustness was examined by introducing controlled variations to instrumental acquisition parameters, including minor adjustments to spectral bandwidth, scan rate, and cuvette positioning. Specificity was verified through evaluation of potential spectral interference from formulation excipients, plasma and co-formulated matrix constituents to ensure selective analyte quantification.

### Elliptical joint confidence region analysis

Comparative statistical appraisal of the developed calibration models was conducted using the Elliptical Joint Confidence Region (EJCR) approach^27,28^. This multivariate inferential procedure evaluates the joint uncertainty associated with regression slope and intercept parameters derived from predicted versus reference concentration relationships. Unlike univariate diagnostics that independently examine proportional or constant error, EJCR provides a simultaneous two-parameter confidence assessment within a bidimensional statistical space. Confidence regions were constructed at the 95% probability level based on the variance–covariance matrix of the estimated regression coefficients obtained from external validation predictions. For each analyte, regression functions generated from PCR, FA-PLS, and MCR-ALS were subjected to EJCR computation. The resulting confidence ellipses were projected relative to the theoretical ideality coordinate defined by unit slope and zero intercept. Ellipse construction and visualization were performed using MATLAB statistical routines, where eigenvalue decomposition of the covariance matrix defined the major and minor ellipse axes and their angular orientation. This framework enabled simultaneous assessment of proportional deviation and constant bias within a unified inferential model. EJCR analysis was applied as an adjunct statistical validation tool alongside conventional multivariate error diagnostics to provide a comprehensive structural evaluation of calibration model behavior.

### Assay of pharmaceutical formulation

The developed spectrophotometric–chemometric procedure was applied to the quantitative determination of CFPM-TAZO in CEFE-MAX™ powder for injection. An accurately weighed portion of the reconstituted powder equivalent to 20 mg CFPM and the corresponding amount of TAZO was quantitatively transferred into a 250-mL volumetric flask. Extraction was performed using ultra-pure water with ultrasonic agitation for 30 min at ambient temperature to ensure complete dissolution of the active constituents. The solution was allowed to equilibrate to room temperature and diluted to volume with water. The resulting solution was filtered through a 0.45 μm membrane filter to remove undissolved excipients and particulate matter. Appropriate aliquots of the filtrate were further diluted with water to obtain final concentrations within the validated analytical domains (1.00–17.00 µg mL^–1^ for CFPM and 1.00–9.00 µg mL^–1^ for TAZO). Absorption spectra were recorded over the selected wavelength interval (210–350 nm) under the predefined instrumental conditions. The processed spectral data were subsequently subjected to the constructed chemometric models for concentration estimation. Matrix validation was performed using the standard addition protocol at three fortification levels corresponding to 80%, 100%, and 120% of the labeled claim. Each level was analyzed in sextuplicate. Quantitative performance was assessed using recovery percentage, relative standard deviation (RSD), and calculated bias in accordance with pharmaceutical validation practices.

### Plasma sample preparation and application to chemometric models

The applicability of the developed spectrophotometric–chemometric platform to biological matrices was evaluated using human plasma. Quantification was performed using the same external calibration models established for pharmaceutical analysis, without employing matrix-matched calibration sets, to assess model robustness and calibration transferability under complex biological conditions.

Drug-free human plasma samples were stored under refrigerated conditions and equilibrated to room temperature prior to analysis. Aliquots of blank plasma (1.0 mL) were spiked with appropriate volumes of working standard solutions of CFPM and TAZO to achieve final concentrations within the validated ranges. Protein precipitation was induced by adding 3.0 mL of cold methanol (1:3, v/v). The mixture was vortexed for 2 min and kept at 4 °C for 15 min to ensure complete protein denaturation and aggregation. Phase separation was achieved by centrifugation at 7000 rpm for 10 min. The clear supernatant was collected, diluted with ultra-pure water as needed, and filtered through a 0.22 μm membrane filter prior to spectral acquisition. Absorption spectra were recorded over the selected wavelength range (210–350 nm) under identical instrumental conditions used for calibration, and the resulting spectral data were processed using the previously developed chemometric models. Matrix effects were evaluated separately using post-extraction spiked plasma samples. Blank plasma was subjected to the same protein precipitation procedure, after which known amounts of CFPM and TAZO were added to the resulting extracts at three concentration levels. Matrix factor (MF) values were calculated by comparing concentrations predicted in post-extraction spiked plasma with those obtained from corresponding neat aqueous standards at equivalent nominal concentrations.

## Results and discussion

### Chemometric model construction and spectral data architecture

#### Multivariate calibration rationale and spectral domain conditioning

The zero-order absorption spectra of CFPM-TAZO (Fig. 1) demonstrate extensive band superimposition across the ultraviolet region, particularly below 250 nm. TAZO exhibits a dominant high-intensity absorption band centered near 211 nm, attributable to π→π* transitions within its β-lactam–thiazole conjugated system. CFPM displays multiple overlapping bands, including a principal absorption region around 270–280 nm associated with its aminothiazolyl and methoxyimino chromophoric moieties.

The pronounced spectral overlap within the 210–250 nm interval results in strong collinearity between absorbance variables, rendering conventional univariate approaches inadequate for selective quantification. Direct measurement at λmax, derivative spectrophotometry, ratio-based manipulation, and dual-wavelength strategies were preliminarily evaluated but failed to provide stable analytical windows free from mutual interference. The absence of isoabsorptive or interference-free wavelengths further confirmed the necessity for multivariate resolution.

Comprehensive spectral acquisition was initially performed over 200–400 nm. Following systematic evaluation of signal-to-noise behavior, solvent absorption contribution, and photometric stability, the operative analytical window was restricted to 210–350 nm at 1 nm resolution, generating 141 wavelength variables per sample. Regions below 210 nm were excluded due to elevated solvent background and increased instrumental noise, whereas absorbance above 350 nm approached baseline levels and contributed negligible discriminatory information. The finalized spectral matrix therefore retained full chromophoric representation for both analytes while eliminating non-informative regions. This conditioning step ensured numerical stability, minimized redundant variance, and provided an optimized dataset for subsequent multivariate calibration modeling.

#### Calibration matrix construction via Brereton multilevel design

The calibration matrix was structured according to Brereton multilevel experimental design principles to ensure uniform population of the bidimensional concentration space for CFPM and TAZO. This design strategy enables systematic distribution of calibration samples while preserving statistical orthogonality within binary mixture systems.

A total of 25 calibration mixtures were generated using a five-level factorial arrangement (− 2, − 1, 0, + 1, +2) applied independently to each analyte. Concentration limits were defined within the validated analytical domains, spanning 1.00–17.00 µg mL^–1^ for CFPM and 1.00–9.00 µg mL^–1^ for TAZO. Central design coordinates (level 0) corresponded to 9 µg mL^–1^ and 5.0 µg mL^–1^ for CFPM and TAZO, respectively.

The multilevel grid incorporated axial, intermediate, and centroid mixtures to provide homogeneous coverage across low, mid, and high concentration regions. Such spatial distribution enhances calibration stability, mitigates leverage clustering, and improves latent variable extraction during chemometric modeling. All calibration compositions and concentration coordinates are detailed in Table 1.

**Table 1:** Five-level, five-factor experimental design comprising 25 calibration and 13 validation mixtures used for chemometric model development and evaluation.

Mix number	Calibration set (µg mL^–1^)	
	CFPM	TAZO
1.	9	5
2.	9	1
3.	1	1
4.	1	9
5.	17	3
6.	5	9
7.	17	5
8.	9	3
9.	5	3
10.	5	7
11.	13	9
12.	17	7
13.	13	5
14.	9	9
15.	17	9
16.	17	1
17.	1	7
18.	13	1
19.	1	5
20.	9	7
21.	13	7
22.	13	3
23.	5	1
24.	1	3
25.	5	5

Mix number	Validation set (µg mL^–1^)	
	CFPM	TAZO
1.	16	2
2.	10	6
3.	3	2
4.	12	2
5.	8	7
6.	2	6
7.	6	9
8.	7	4
9.	14	1
10.	15	7
11.	4	8
12.	17	4
13.	12	5

#### Validation set optimization via Fedorov exchange design

**Fig. 2:**
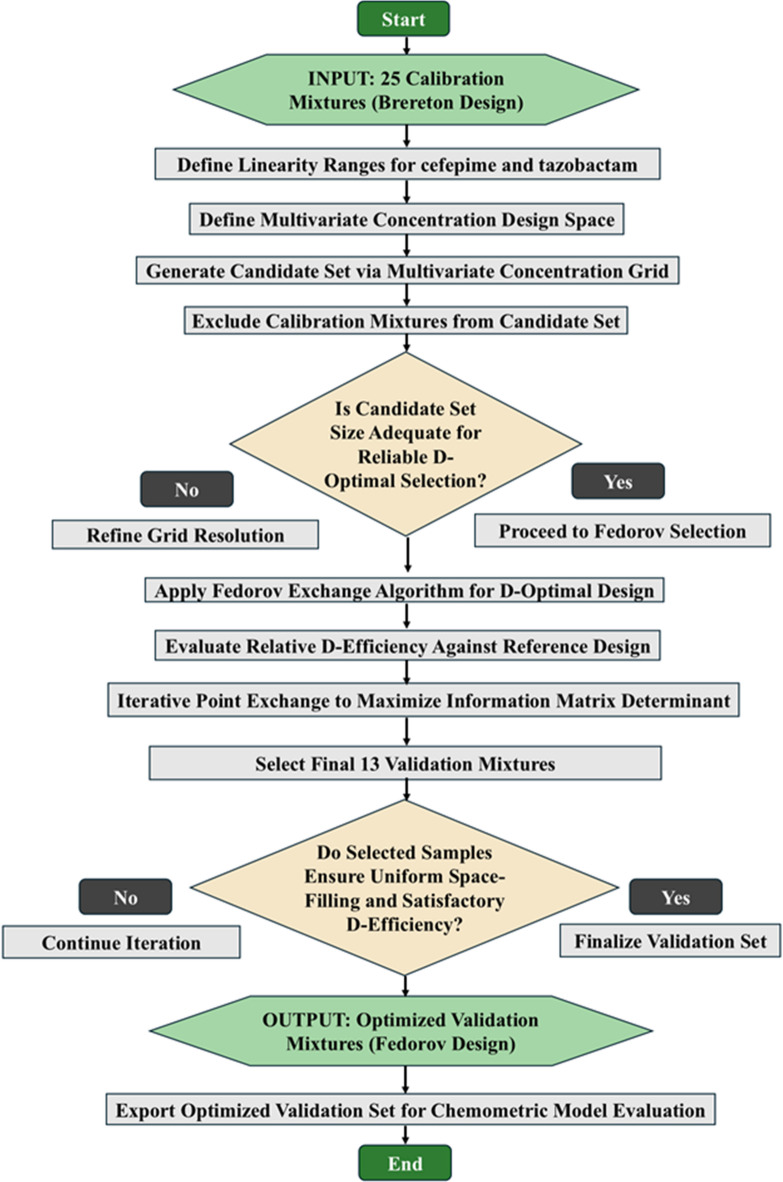
Workflow of the Fedorov exchange algorithm employed for selecting an optimized validation set.

External validation architecture was established through implementation of the Fedorov exchange algorithm to derive an information-optimal subset of mixture compositions within the predefined CFPM-TAZO concentration domain. The optimization workflow is schematically illustrated in Fig. 2, which delineates the sequential computational logic governing validation subset construction. The process was initiated by defining multidimensional design boundaries based on the validated linearity intervals of both analytes. A comprehensive candidate pool was subsequently generated via multilevel grid enumeration spanning the entire bidimensional concentration space. Calibration coordinates were excluded from this pool to preserve statistical independence between model training and external validation phases.

Exchange optimization was then executed according to D-optimality criteria. At each iteration, candidate validation points were algorithmically substituted and re-evaluated to maximize the determinant of the Fisher information matrix. This determinant maximization serves to minimize the joint confidence volume of regression parameters, thereby improving predictive precision and reducing extrapolation uncertainty across the design space. Convergence was reached once determinant stabilization was achieved within predefined tolerance thresholds.

**Fig. 3:**
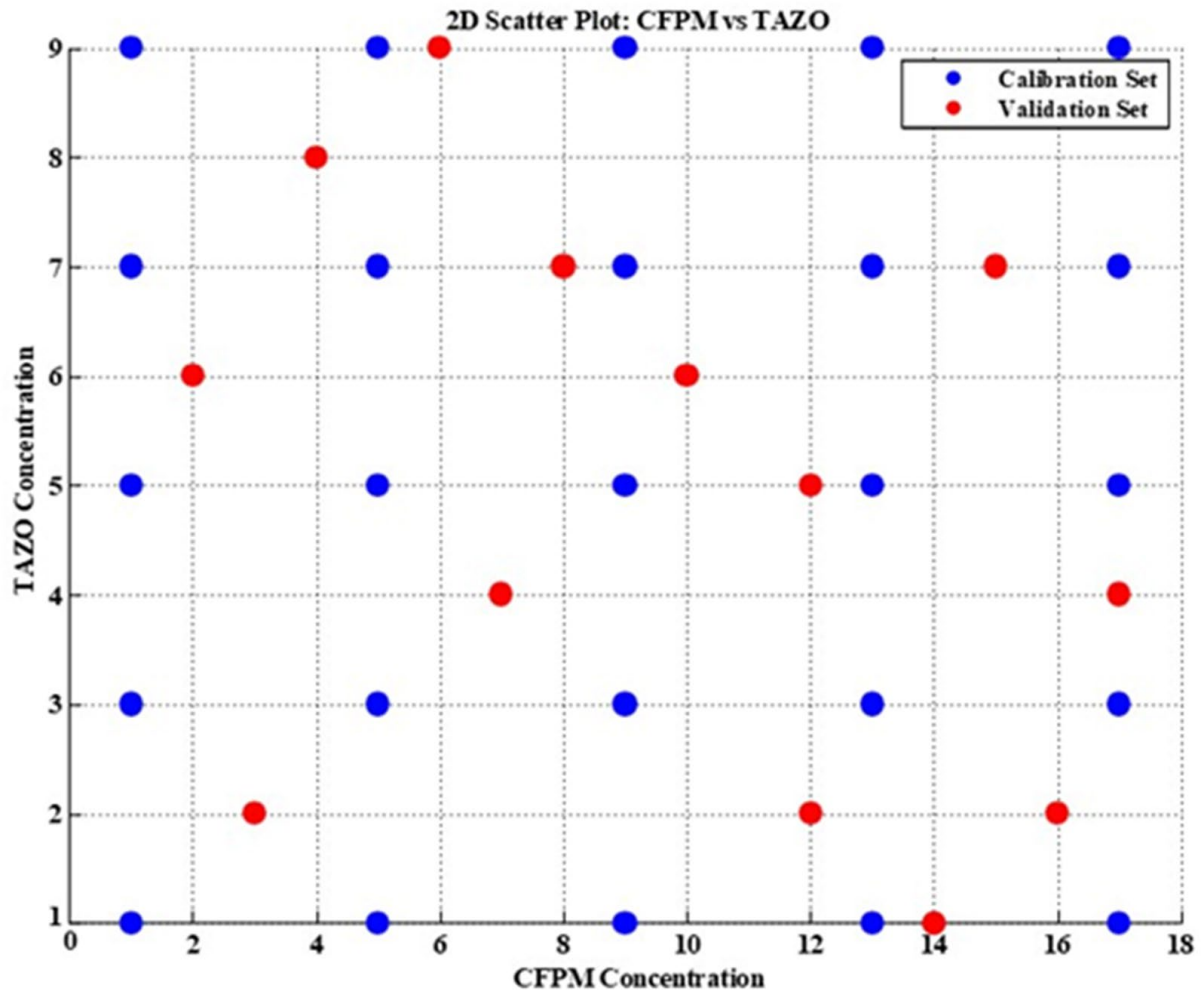
Two-dimensional scatter plot of the Fedorov-designed validation set, illustrating optimal space-filling distribution.

Geometric distribution diagnostics of the finalized validation subset are presented in Fig. 3. The two-dimensional scatter projection demonstrates homogeneous spatial dispersion of validation samples relative to the calibration matrix. Validation points occupy peripheral, intermediate, and centroidal regions, ensuring that predictive performance is interrogated across the full analytical domain rather than confined to localized concentration zones. The absence of clustering or leverage accumulation confirms that the exchange algorithm successfully enforced spatial decorrelation.

**Fig. 4:**
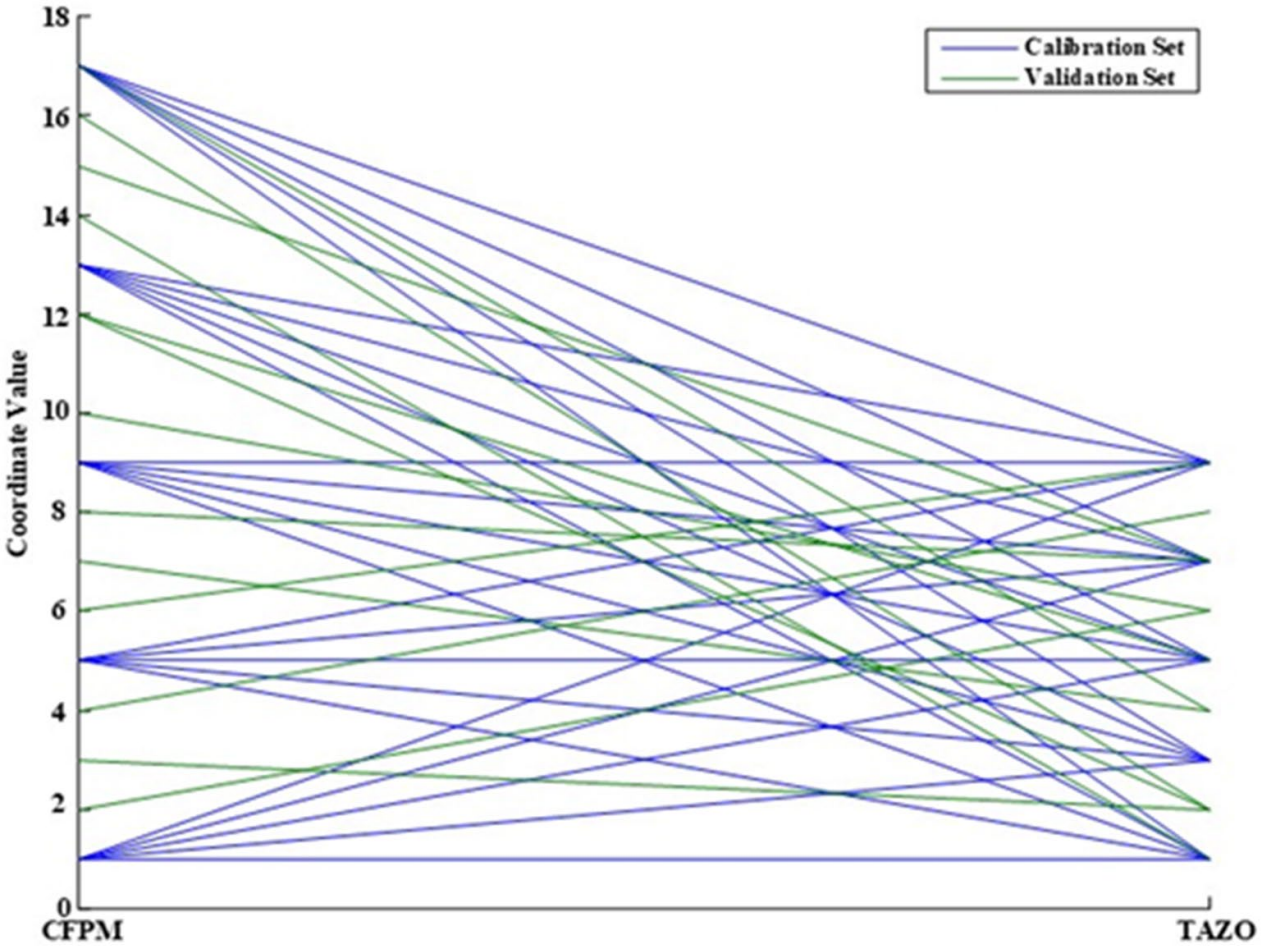
Parallel coordinate plot comparing analyte concentration distributions in the calibration and validation sets.

Complementary compositional mapping using parallel-coordinate visualization (Fig. 4) further substantiates validation representativeness. Each polyline trajectory corresponds to a mixture composition projected simultaneously across both analyte axes. The interwoven yet non-redundant trajectory pattern indicates that validation samples traverse concentration gradients analogous to calibration mixtures while maintaining structural independence. This duality ensures that predictive assessment reflects realistic interpolation and boundary behavior without statistical redundancy. The integrated Brereton–Fedorov design framework therefore establishes a bifurcated experimental architecture in which calibration density and validation informativeness are independently optimized. Such partitioned optimization enhances model generalizability by ensuring that performance evaluation is conducted on mixtures selected for maximal information content rather than arbitrary distribution.

### Multivariate calibration model development and comparative evaluation

#### Principal component regression modeling

PCR was implemented as the baseline linear multivariate calibration framework to model the spectral–concentration relationship within the binary system^29^. The approach integrates principal component decomposition with subsequent regression of concentration vectors onto orthogonal latent variables, thereby alleviating multicollinearity inherent to overlapping UV absorption data.

Spectral matrices acquired over the optimized wavelength interval (210–350 nm; 141 variables) were subjected to principal component transformation. This orthogonalization compresses covariance structure into a reduced latent space while suppressing stochastic noise and instrumental variance contributions. Prior to decomposition, spectra were mean-centered and variance-scaled to standardize variable weighting and enhance numerical conditioning.

Model dimensionality was established through leave-one-out cross-validation, where prediction residuals were monitored as a function of latent variable (LV) expansion. The RMSECV profiles presented in Fig. S2A exhibit a steep error decline between LV_1_ and LV_2_ for both analytes, reflecting rapid capture of chemically relevant variance. Beyond the third LV, error stabilization is observed, indicating diminishing returns in predictive information and the onset of variance redundancy. Accordingly, three latent variables were retained for both CFPM and TAZO as the optimal model complexity. This dimensionality provided an adequate balance between variance representation and generalization capacity without introducing overparameterization. Inspection of loading structures revealed that principal variance contributions originated predominantly from the 210–250 nm region, where combined chromophoric absorption of both analytes is most intense. Secondary contributions were distributed across mid-UV regions corresponding to auxiliary electronic transitions. These loading distributions confirm that PCR modeling effectively exploits overlapped spectral information rather than relying on isolated wavelength maxima.

#### Firefly Algorithm–assisted PLS variable optimization

To enhance regression selectivity beyond full-spectrum modeling, wavelength subset optimization was implemented using the FA-PLS^23^. This hybrid chemometric framework integrates swarm-intelligence search dynamics with latent-variable calibration to refine spectral dimensionality prior to model construction. Within the FA architecture, each firefly encoded a candidate wavelength subset derived from the preprocessed spectral matrix (210–350 nm; 141 variables). Solution fitness was evaluated using cross-validated prediction residuals, where firefly attractiveness was directly proportional to model predictive performance. Iterative positional updating was governed by attractiveness decay and stochastic randomization parameters, enabling convergence toward spectrally informative regions while suppressing noise-dominated variables. Algorithmic control parameters are summarized in Table S1. Population size was fixed at 25 fireflies for both analytes, with evolutionary propagation conducted over 100 generations. Randomization coefficients (α) were set at 0.25 for cefepime (CFPM) and 0.20 for tazobactam (TAZO), balancing exploratory search behavior and convergence stability. Initial attractiveness (β_0_ = 1.0) permitted unrestricted attraction at minimal inter-firefly distance, whereas absorption coefficients (γ = 0.9 for CFPM; 0.8 for TAZO) regulated light-intensity attenuation across the optimization landscape. Variable-selection outcomes demonstrated substantial dimensionality compression relative to the full spectral matrix. The FA-PLS procedure retained approximately 58% of the original variables for CFPM (82 wavelengths) and 66% for TAZO (93 wavelengths), corresponding to spectral reductions of 42% and 34%, respectively. These retention ratios reflect the adaptive response of the algorithm to analyte-specific spectral complexity and overlap structure. Optimized PLS dimensionality was determined through cross-validation profiling. The RMSECV trajectories illustrated in Fig. S2B reveal a sharp decline in prediction residuals upon transition from the first to the second latent variable, followed by error stabilization at higher latent orders. This curvature indicates that the principal covariance structure is effectively captured within the first two latent variables, with subsequent components contributing predominantly stochastic variance. Accordingly, two latent variables were retained for both analytes in the finalized FA-PLS models, providing reduced model complexity relative to PCR while preserving essential predictive information. Mapping of retained wavelength domains indicated primary localization within the 210–260 nm interval, corresponding to the dominant overlapping chromophoric absorption of the binary system, with auxiliary contributions extending into mid-UV transition regions.

#### Multivariate curve resolution–ALS spectral deconvolution

The MCR-ALS was implemented as a bilinear resolution framework to decompose the overlapped spectral matrix of CFPM and TAZO into their corresponding pure concentration and spectral profiles. Unlike regression-based approaches, MCR-ALS operates through iterative matrix factorization without requiring predefined calibration vectors, enabling direct extraction of chemically meaningful information from mixture datasets^30,31^.

**Fig. 5:**
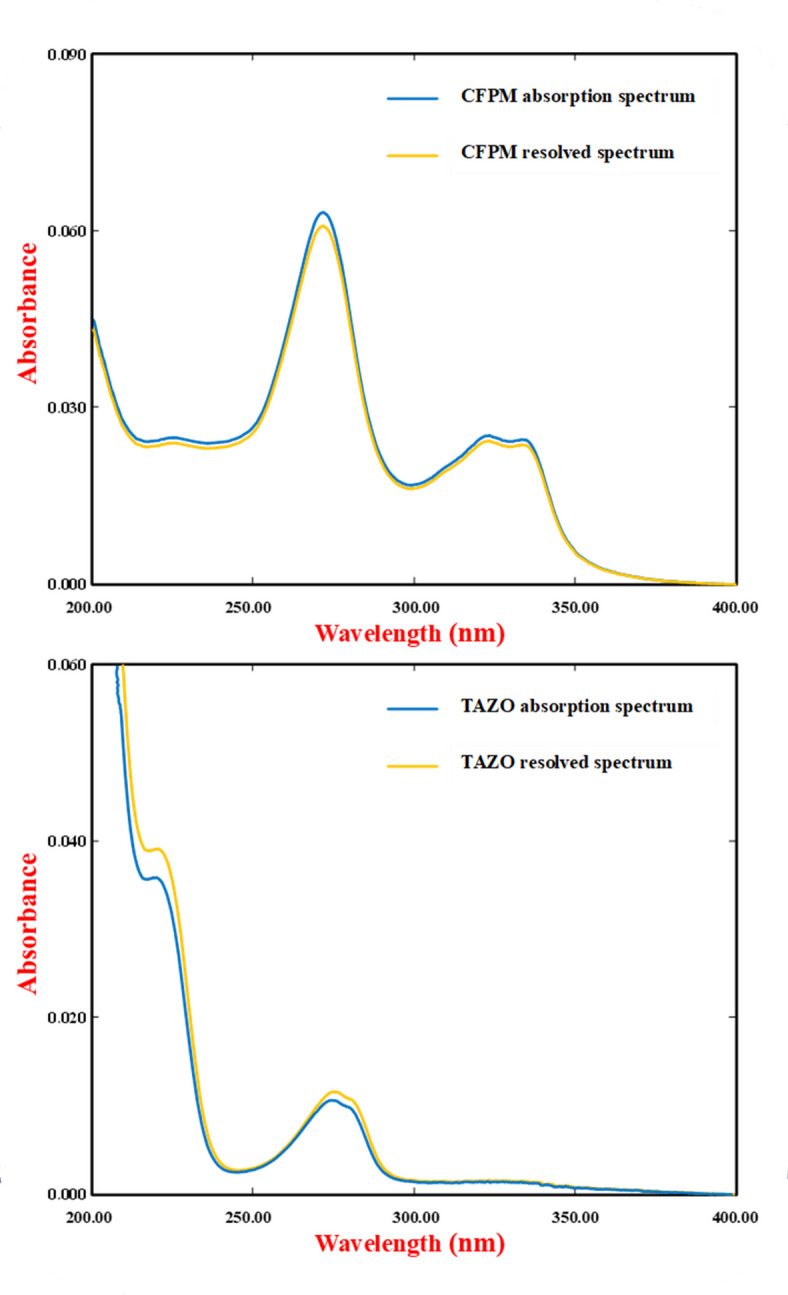
Absorption spectra of the analyzed mixtures and pure component spectra resolved by MCR-ALS.

Model construction was performed using the calibration spectral matrix derived from the 25-mixture Brereton design. System rank estimation was conducted via Evolving Factor Analysis (EFA), where eigenvalue progression indicated the presence of two dominant factors corresponding to the binary analyte system. No additional latent contributions were detected, confirming the absence of spectrally active interferents within the analytical domain. Alternating least squares optimization was subsequently executed under physicochemical constraints, including non-negativity applied to both concentration and spectral matrices and closure constraints imposed on mixture compositions. Iterative refinement proceeded until convergence criteria were satisfied based on residual stabilization thresholds. The resolved pure spectral estimates are presented in Fig. 5. Recovered CFPM profiles exhibit characteristic absorption features within the 260–280 nm region, accompanied by secondary transitions extending toward the mid-UV domain. The resolved TAZO spectrum displays dominant high-intensity absorption below 230 nm with a subsidiary band near 280 nm, consistent with its β-lactam–sulfonyl chromophoric structure. Close superimposition between resolved and reference spectra confirms the fidelity of the bilinear decomposition. Spectral reconstruction across the full wavelength domain demonstrates effective resolution of the severe overlap observed below 250 nm, indicating that the applied constraint set was sufficient to recover chemically interpretable component signatures without rotational ambiguity.

### Chemometric model validation and comparative performance

#### External predictive validation via Fedorov-optimized design

External predictive performance was evaluated using thirteen validation mixtures selected through the Fedorov exchange algorithm to ensure uniform spatial coverage across the bidimensional concentration domain. The validation matrix remained statistically independent from the calibration design while spanning the complete analytical working ranges of CFPM and TAZO, as shown in (Table 1). This architecture enabled unbiased interrogation of model generalizability under both central and boundary concentration conditions.

#### Comparative quantitative performance of chemometric models

Analytical performance metrics derived from calibration and validation datasets are summarized in (Table 2). Comparative evaluation revealed progressive enhancement in predictive fidelity upon transition from linear regression modeling to learning-assisted optimization and bilinear resolution frameworks.

**Table 2:** Analytical performance of the proposed chemometric models for the determination of TAZO and CFPM in calibration and validation sample sets.

		PCR		FA-PLS		MCR-ALS	
		CFPM	TAZO	CFPM	TAZO	CFPM	TAZO
Calibration set	MEAN	99.32	99.56	99.71	99.83	100.12	100.05
	SD	0.618	0.689	0.487	0.532	0.352	0.398
	%RSD	0.622	0.692	0.489	0.533	0.352	0.398
	RMSEC^(a)^	0.326	0.301	0.248	0.221	0.186	0.163
Validation set	MEAN	99.18	99.42	99.64	99.79	100.08	100.02
	SD	0.673	0.724	0.512	0.558	0.381	0.421
	%RSD	0.679	0.728	0.514	0.559	0.381	0.421
	RMSEP^(b)^	0.371	0.345	0.281	0.257	0.214	0.192

^a^ Root Mean Square Error of calibration.

^b^ Root Mean Square Error of predication.

PCR demonstrated acceptable baseline predictive capability, yielding RMSEP values of 0.371 µg mL^–1^ for CFPM and 0.345 µg mL^–1^ for TAZO. Calibration trueness remained within pharmacopeial acceptance limits, with mean recoveries of 99.32% and 99.56%, respectively. However, comparatively elevated RMSEC and prediction residuals reflect the intrinsic limitation of full-spectrum linear modeling in highly collinear spectral systems.

Integration of FA variable selection produced measurable improvements in model precision. Dimensionality refinement reduced redundant spectral variance, yielding RMSEP values of 0.281 µg mL^–1^ for CFPM and 0.257 µg mL^–1^ for TAZO. Calibration recoveries (99.71% and 99.83%) and reduced %RSD values further confirm enhanced regression stability following wavelength subset optimization.

MCR-ALS exhibited the highest predictive performance among the evaluated models. External validation errors were minimized to 0.214 µg mL^–1^ for CFPM and 0.192 µg mL^–1^ for TAZO, accompanied by near-unity recovery means (100.08% and 100.02%). The reduced RMSEC and prediction dispersion reflect the algorithm’s capacity to resolve pure component contributions before quantification, thereby mitigating covariance interference inherent in overlapped spectral matrices.

#### Comprehensive analytical method validation

Method validation of the proposed spectrophotometric–chemometric platform was performed in accordance with ICH Q2(R1) guidelines^32^, encompassing sensitivity, trueness, precision, and predictive reliability. Validation parameters for all models are summarized in Table S2.

Sensitivity was evaluated using the net analyte signal (NAS) approach. LOD for CFPM were 0.0863 µg mL^–1^ (PCR), 0.0648 µg mL^–1^ (FA-PLS), and 0.0487 µg mL^–1^ (MCR-ALS). Corresponding values for TAZO were 0.0724, 0.0529, and 0.0396 µg mL^–1^, respectively. LOQ followed the same performance hierarchy, ranging from 0.2615 to 0.1476 µg mL^–1^ for CFPM and 0.2194 to 0.1200 µg mL^–1^ for TAZO. These detection thresholds confirm adequate method sensitivity for dosage form and bioanalytical applications.

Calibration and predictive performance were further assessed using residual error metrics, including (RMSEC), (RMSEP), (RRMSEP), (BCRMSEP), and (SEC). RMSEC values indicated progressive improvement in calibration fitting fidelity from PCR to FA-PLS and ultimately MCR-ALS, reflecting enhanced variance capture following variable optimization and bilinear resolution. External predictive reliability, expressed via RMSEP, exhibited the same hierarchical trend, confirming that reduced calibration error translated into improved performance on independent validation mixtures. RRMSEP remained below 2.5% for all models, with the lowest values obtained using MCR-ALS, indicating superior proportional prediction accuracy. Bias-corrected RMSEP values were consistently lower than uncorrected RMSEP estimates, demonstrating negligible systematic prediction offset. Likewise, reduced SEC values for FA-PLS and MCR-ALS confirm enhanced calibration stability compared with full-spectrum PCR modeling.

Method accuracy was evaluated through recovery studies across the validated analytical ranges. Mean percentage recoveries ranged from 99.32% to 100.12% for both analytes. The MCR-ALS model exhibited the closest agreement with theoretical concentrations, yielding recoveries of 100.12% for CFPM and 100.05% for TAZO, with minimal dispersion.

Precision was assessed under repeatability and intermediate precision conditions. Intra-day %RSD values did not exceed 0.724% for PCR, 0.548% for FA-PLS, and 0.402% for MCR-ALS. Inter-day precision remained below 0.832% across all models and analytes. The reduced variability observed for FA-PLS and MCR-ALS reflects improved signal discrimination and covariance handling relative to full-spectrum regression. Overall, all evaluated validation parameters satisfied pharmacopeial acceptance criteria, confirming the reliability of the developed chemometric platform for quantitative determination of CFPM and TAZO in pharmaceutical and plasma matrices.

#### Statistical comparative evaluation

##### Student’s t-test accuracy verification

Method accuracy was further examined using Student’s t-test comparing experimental recoveries against theoretical 100% values^33^. Calculated t-values for all analytes and models remained below the tabulated limits (t_0.025,12_ = 2.179; t_0.01,12_ = 2.681), confirming absence of significant systematic bias (Table S2). Among the evaluated frameworks, MCR-ALS exhibited the lowest t-statistics, reflecting the highest recovery consistency and minimal proportional deviation. PCR demonstrated comparatively higher t-values, consistent with its reliance on full-spectrum regression under conditions of pronounced spectral collinearity.

##### One-way ANOVA assessment

Inter-model performance variability was statistically interrogated using one-way analysis of variance (ANOVA) at a 95% confidence level (Table S3). For CFPM, the calculated F-value (1.38) remained below the critical threshold (Fcrit = 3.89), with a corresponding p-value of 0.279. Similarly, TAZO exhibited F = 1.11 with *p* = 0.346.

Because Fcalculated < Fcritical and *p* > 0.05 in both cases, no statistically significant difference was observed among the evaluated chemometric models. These findings indicate that all models provide comparable quantitative performance within pharmaceutical matrices, despite observable numerical differences in prediction error metrics.

##### Elliptical Joint Confidence Region (EJCR) analysis

To provide a rigorous multivariate assessment of model trueness and proportional bias, EJCR analysis was performed for the regression of predicted versus reference concentrations. Unlike univariate metrics (e.g., RMSEP or r^2^), EJCR simultaneously evaluates the joint confidence limits of slope and intercept, enabling integrated assessment of constant and proportional systematic error within a unified statistical framework. The 95% joint confidence ellipses for PCR, FA-PLS, and MCR-ALS are illustrated in (Fig. S3) for both drugs. The theoretical ideal analytical performance is represented by the coordinate (slope = 1.0, intercept = 0.0).

For both analytes, PCR exhibited the largest confidence ellipses, with centers displaced from the ideal point along both axes. The broader ellipse area reflects increased uncertainty in slope and intercept estimation, while the positional shift indicates the presence of minor proportional and constant bias components. These observations are consistent with the higher RMSEP and RRMSEP values obtained for PCR.

The FA-PLS model demonstrated reduced ellipse dimensions relative to PCR, indicating improved precision in regression parameter estimation. The ellipse centroid was positioned closer to the ideal coordinate, suggesting diminished systematic deviation following wavelength subset optimization. The reduced spread confirms improved covariance handling compared with full-spectrum regression.

MCR-ALS produced the smallest and most compact confidence regions for both analytes. The ellipses were centered in close proximity to the theoretical ideal coordinate and encompassed it within the 95% confidence limits. The reduced area of the confidence region reflects minimal uncertainty in slope–intercept estimation and confirms superior regression stability.

The progressive contraction of ellipse area from PCR to FA-PLS and finally MCR-ALS mirrors the hierarchy observed in residual diagnostics (RMSEP, BCRMSEP) and precision metrics. While one-way ANOVA indicated no statistically significant inter-model difference at the 95% confidence level, EJCR analysis provides enhanced discriminatory resolution by evaluating regression parameter covariance rather than mean recovery alone.

The graphical evidence therefore substantiates that MCR-ALS achieves the lowest combined proportional and constant error among the evaluated chemometric strategies. This behavior is consistent with its bilinear decomposition mechanism, which isolates pure component contributions prior to quantification, thereby reducing covariance-induced bias.

### Application to pharmaceutical product (CEFE-MAX™)

The practical applicability of the proposed spectrophotometric–chemometric platform was evaluated through the direct analysis of GEFE-MAX™ powder for injection. Quantitative results obtained are summarized in (Table 3).

**Table 3:** Determination of TAZO and CFPM in tablet formulations using the proposed chemometric methods and comparison with the standard addition approach.

Preparation		%Recovery ± %RSD ^(a)^		
		PCR	FA-PLS	MCR-ALS
CFPM	Direct tablet analysis	99.33 ± 1.44	99.68 ± 1.12	100.10 ± 0.86
	Standard addition	99.82 ± 1.21	100.05 ± 0.94	100.28 ± 0.71
TAZO	Direct tablet analysis	99.18 ± 1.61	99.54 ± 1.19	99.96 ± 0.91
	Standard addition	99.63 ± 1.28	99.88 ± 0.96	100.14 ± 0.74

^a^ Average of six determinations.

#### Direct assay performance

For CFPM, direct determination yielded mean recoveries of 99.33%, 99.68%, and 100.10% using PCR, FA-PLS, and MCR-ALS, respectively. For TAZO, corresponding recoveries were 99.18%, 99.54%, and 99.96%. All results fall within pharmacopeial acceptance limits (98–102%), confirming the suitability of the developed models for routine quality control analysis of the combined formulation. Relative standard deviation values remained below 1.61% for all analytes and models, demonstrating satisfactory precision in the presence of formulation excipients.

#### Standard addition verification

To further evaluate matrix effects and confirm method trueness, standard addition experiments were performed at multiple fortification levels. Recovery values ranged from 99.82% to 100.28% for CFPM and from 99.63% to 100.14% for TAZO. The close agreement between direct assay and standard addition results indicates negligible matrix interference and confirms that excipient components do not significantly influence spectral resolution or chemometric prediction.

#### Comparative model performance in dosage matrix

Among the evaluated models, MCR-ALS exhibited the lowest dispersion in both direct and spiked analyses (%RSD ≤ 0.91%), reflecting superior stability under real formulation conditions. FA-PLS showed intermediate performance, while PCR displayed slightly higher variability, consistent with observations from validation and residual error analyses.

The observed hierarchy mirrors the progressive enhancement in signal discrimination capability from full-spectrum regression to variable-optimized modeling and ultimately to bilinear spectral resolution.

#### Practical implications

The ability to accurately quantify both analytes in a commercial dosage form without chromatographic separation confirms that the proposed UV–chemometric methodology provides:

- Reliable multicomponent resolution despite spectral overlap.
- Minimal susceptibility to excipient interference.
- Precision compatible with regulatory quality control standards.
- Rapid and solvent-minimized analysis.

These findings validate the method’s suitability for routine pharmaceutical quality assurance applications.

### Application to human plasma and calibration transferability assessment

The applicability of the developed spectrophotometric–chemometric platform to biological matrices was investigated using spiked human plasma samples. Blank plasma was fortified with CFPM and TAZO prior to protein precipitation; thus, the recoveries reported in Table 4 represent overall process efficiency, including extraction recovery, residual matrix effects, and chemometric prediction performance. Quantification was performed using the same external calibration models established for pharmaceutical analysis, without the use of matrix-matched calibration sets. This approach enabled assessment of model robustness and calibration transferability under physiologically relevant conditions.

**Table 4:** Determination of CFPM and TAZO in Human Plasma Samples.

Analyte	Model	Spiked level (µg mL^–1^)	%Recovery ± %RSD ^(a)^
CFPM	PCR	5.0	94.92 ± 2.54
		10.0	95.48 ± 2.18
		15.0	96.03 ± 1.96
	FA-PLS	5.0	96.08 ± 2.07
		10.0	96.71 ± 1.79
		15.0	97.26 ± 1.58
	MCR-ALS	5.0	96.92 ± 1.71
		10.0	97.54 ± 1.49
		15.0	98.01 ± 1.32
TAZO	PCR	3.0	94.38 ± 2.73
		5.0	95.01 ± 2.29
		7.0	95.62 ± 2.04
	FA-PLS	3.0	95.67 ± 2.14
		5.0	96.29 ± 1.86
		7.0	96.88 ± 1.67
	MCR-ALS	3.0	96.53 ± 1.78
		5.0	97.18 ± 1.55
		7.0	97.72 ± 1.38

^a^ Average of six determinations.

#### Recovery performance in plasma

For CFPM, recoveries ranged from 94.92% to 98.01% across the investigated concentration levels and chemometric models. For TAZO, recoveries ranged from 94.38% to 97.72%. These values were slightly lower than those obtained for the pharmaceutical formulation, which is expected given the additional contributions of sample preparation losses and residual matrix-induced signal suppression in plasma analysis.

Overall, the observed recoveries indicate efficient protein precipitation and satisfactory analyte extraction. Despite the complexity of the biological matrix and the absence of matrix-matched calibration, all results remained within acceptable limits for exploratory bioanalytical applications and screening-oriented therapeutic monitoring.

#### Matrix effect evaluation

Matrix effects were evaluated using post-extraction spiked plasma samples analyzed with the optimized MCR-ALS model (Table S4). Matrix factor values ranged from 0.9780 to 0.9873 for CFPM and from 0.9765 to 0.9842 for TAZO, corresponding to matrix effect values between − 2.35% and − 1.27%. The negative values indicate slight signal suppression, whereby analytical responses in plasma extracts were marginally lower than those obtained from neat aqueous standards. Given that signal suppression did not exceed 2.35% and all matrix factor values were close to unity, endogenous plasma constituents were considered to have a negligible impact on analyte quantification.

#### Precision in biological matrix

The %RSD values did not exceed 2.73% for any analyte, concentration level, or chemometric model, demonstrating satisfactory repeatability under biological conditions. As observed throughout the validation study, model performance followed the order:

$${\text{MCR - ALS }} > {\text{ FA - PLS }} > {\text{ PCR}}$$

MCR-ALS consistently provided the highest recoveries and lowest variability, highlighting its superior ability to resolve analyte-specific spectral information from residual plasma background contributions.

#### Calibration transferability and matrix tolerance

The successful application of external calibration models to plasma samples demonstrates satisfactory calibration transferability. The combination of acceptable recoveries and minimal matrix effects confirms that the developed chemometric models retain predictive stability even in the absence of matrix-matched calibration. This finding is particularly significant given the pronounced spectral overlap between the analytes and endogenous plasma components in the deep UV region.

#### Practical implications

Although the proposed method is not intended to replace chromatographic bioanalytical techniques for ultra-trace quantification and comprehensive pharmacokinetic studies, the results demonstrate the practical utility of the developed green UV–chemometric platform for rapid plasma analysis. The method offers several advantages, including minimal sample preparation based on a simple protein precipitation step, low solvent consumption, reduced analytical cost, and a substantially lower environmental footprint compared with conventional chromatographic procedures.

From a pharmacokinetic perspective, CFPM exhibits linear pharmacokinetics, low plasma protein binding (~ 20%), minimal metabolism, and predominant renal excretion as unchanged drug, with an elimination half-life of approximately 2 h. Reported peak plasma concentrations following therapeutic intravenous administration are typically around 30.7 µg mL^–1^^34^, although higher values may occur depending on dose and infusion regimen. TAZO also undergoes minimal metabolism, shows limited plasma protein binding, and is rapidly eliminated via the kidneys, with an elimination half-life of approximately 1 h and reported peak plasma concentrations near 10 µg mL^–1^^35^.

The validated concentration ranges of the proposed method (1–17 µg mL^–1^ for CFPM and 1–9 µg mL^–1^ for TAZO) therefore cover clinically relevant levels encountered during post-distribution and elimination phases, as well as diluted plasma samples commonly used in routine analysis. In addition, the low detection limits achieved by the developed models enable reliable quantification of sub-therapeutic concentrations.

Accordingly, the method is well suited for preliminary therapeutic drug monitoring, dose-adjustment screening, stability studies, and rapid plasma analysis in settings where simplicity, cost-effectiveness, and environmental sustainability are prioritized. The successful quantification of both analytes using externally calibrated models, together with the negligible matrix effects (Table S4), further confirms the robustness and calibration transferability of the chemometric framework under biologically complex conditions.

Although validation was performed using fortified blank human plasma rather than samples from treated patients, this approach is widely accepted in early-stage bioanalytical method development. Future application to authentic clinical samples will further confirm the method’s utility in real-world therapeutic monitoring.

Collectively, these findings extend the applicability of the proposed methodology beyond pharmaceutical quality control to rapid, environmentally sustainable plasma analysis while maintaining satisfactory quantitative reliability.

### Sustainability assessment

Greenness and sustainability are related but distinct concepts. Greenness refers specifically to minimizing the environmental impact of analytical procedures through reduced use of hazardous reagents, lower energy consumption, and decreased waste generation. Sustainability is a broader framework that additionally integrates analytical performance, practicality, innovation, economic feasibility, and societal relevance. Accordingly, the proposed methodology was evaluated using complementary assessment tools to capture both dimensions and benchmarked against representative reported methods.

#### Multi-Color Assessment (MA) sustainability profiling

The sustainability of the proposed analytical platform was evaluated using the Multi-Color Assessment (MA) tool, a holistic framework encompassing environmental impact (GEMAM), practical applicability (BAGI), analytical performance (RAPI), and innovation (VIGI), based on a structured 51-question protocol^25^.

The method achieved GEMAM (Greenness) of 90.1%, BAGI (Blueness) of 86.2%, RAPI (Redness) of 87.9%, and VIGI (Violet) of 70.0%, yielding an overall Whiteness Score of 83.6%. This reflects excellent sustainability with a well-balanced analytical profile.

Benchmarking against reported methods demonstrated clear superiority, with higher whiteness than HPLC–UV (62.6%), LC–MS/MS (61.4%), capillary zone electrophoresis (65.6%), and conventional UV spectrophotometry (74.9%). This performance is attributed to the exclusive use of water as solvent, elimination of chromatographic separation, minimal waste generation, and the integration of chemometric modeling with Fedorov-optimized experimental design (Table 5).

**Table 5:**
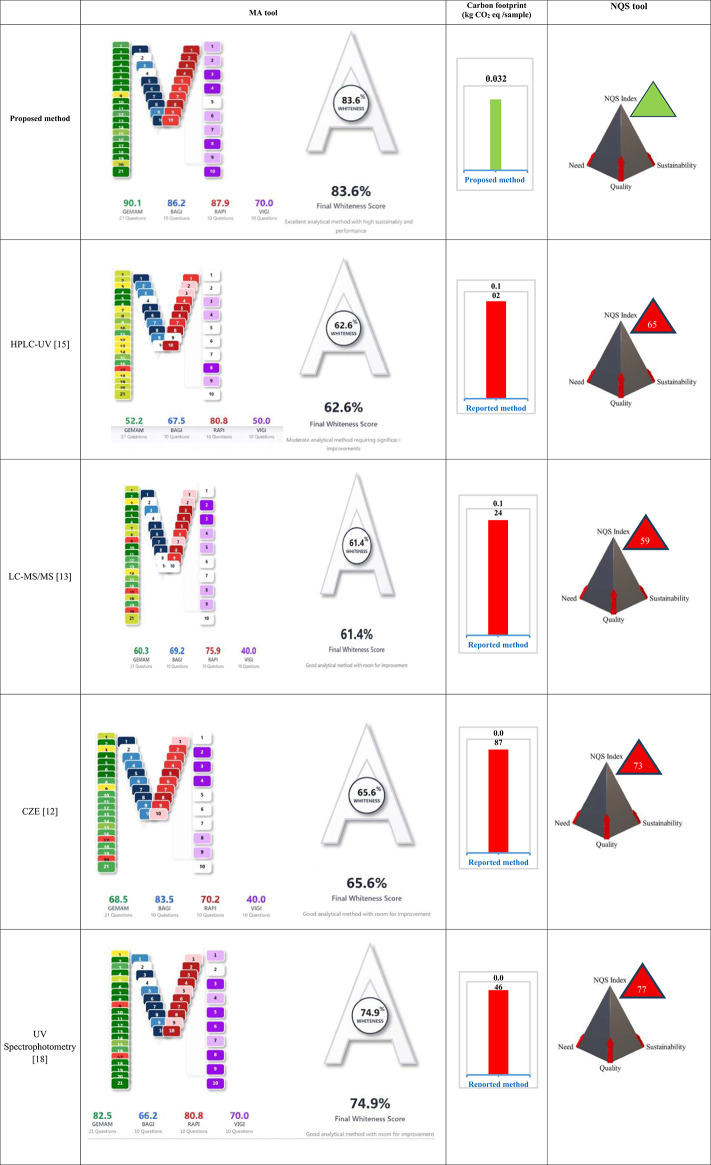
Comprehensive sustainability and performance assessment of the proposed analytical approach.

#### Carbon Footprint Analysis (CFA)

To further substantiate the environmental performance of the proposed UV–chemometric platform, the carbon footprint (CF) was evaluated using an expanded system boundary. This cradle-to-gate assessment accounted for both direct energy consumption and indirect emissions associated with routine sample analysis^36^.

The analysis included electricity usage from the UV–visible spectrophotometer and ultrasonic bath, as well as the environmental impact of methanol production, disposable PVDF filtration units, consumables used during sample handling, and solvent waste management. Impacts from instrument manufacturing and laboratory infrastructure were excluded, as they are amortized over long operational lifetimes and are not representative of per-sample analytical contributions.

The carbon footprint per analysis was calculated according to:

$${\mathrm{C}}{{\mathrm{F}}_{{\mathrm{sample}}}}=\sum \left( {{P_i} \times {t_i} \times E{F_{{\mathrm{elec}}}}} \right)+\sum \left( {{m_j} \times E{F_j}} \right)$$

where ${P_i}$ represents instrument power consumption (kW), ${t_i}$ denotes operating time (h), and $E{F_{elec}}$ corresponds to the electricity emission factor (kg CO_2_-eq kWh^–1^). The second summation accounts for consumable-related emissions, where ${m_j}$ is the mass of each material and $E{F_j}$ its cradle-to-gate emission factor. The total carbon footprint of the proposed method was 0.032 kg CO_2_-eq per sample. This value was substantially lower than those obtained for the compared methods: HPLC-UV: 0.102 kg CO₂-eq per sample, LC-MS/MS: 0.124 kg CO_2_-eq per sample, CZE: 0.087 kg CO_2_-eq per sample, and Conventional UV spectrophotometry: 0.046 kg CO_2_-eq per sample. The approximately three- to four-fold reduction relative to chromatographic methods is primarily attributable to the absence of organic mobile phases, lower instrumental energy consumption, and simplified sample preparation.

#### Need–Quality–Sustainability (NQS) assessment and UN-SDG alignment

To contextualize the developed methodology within a global sustainability framework, the (NQS) Index was applied as an integrated multidimensional evaluation tool^37^. Unlike single-domain assessment approaches, the NQS framework simultaneously examines analytical performance, societal relevance, and environmental responsibility, thereby aligning method evaluation with the principles of the United Nations Sustainable Development Goals (UN-SDGs). The proposed UV–chemometric method achieved an overall NQS score of 90% (Table 5), indicating a high level of balanced sustainability integration across all three domains. The strong performance within the Need dimension reflects the method’s relevance to clinically important antimicrobial analysis, specifically the determination of cefepime–tazobactam in pharmaceutical dosage forms and biological matrices, supporting quality assurance and therapeutic monitoring applications. The Quality dimension score is supported by validated precision, sensitivity, predictive reliability, and robust multivariate performance across calibration, validation, and real-sample investigations. The Sustainability dimension is reinforced by exclusive use of water as solvent, low instrumental energy demand, minimized chemical waste generation, and reduced experimental workload achieved through Fedorov-optimized experimental design.

To further contextualize its societal alignment, the method was mapped against relevant UN-SDGs (Table 6). It contributes to SDG 3 (Good Health and Well-being) through support of antimicrobial quality control and therapeutic monitoring; SDG 4 (Quality Education) by providing a practical platform for teaching green analytical chemistry and chemometric design principles; SDG 7 (Affordable and Clean Energy) through reduced energy requirements relative to chromatographic systems; SDG 9 (Industry, Innovation, and Infrastructure) via implementation of advanced chemometric modeling and optimized experimental design; SDG 12 (Responsible Consumption and Production) by minimizing hazardous solvent usage and analytical waste; and SDG 13 (Climate Action), supported by a low per-sample carbon footprint of 0.032 kg CO₂-equivalent. A triangular NQS visualization (Fig. S4) illustrates the interaction among Need, Quality, and Sustainability dimensions, positioning the developed method near the apex of balanced multidimensional performance. When interpreted alongside (MA) outcomes and carbon footprint analysis, the NQS framework provides convergent evidence that the proposed analytical platform integrates environmental compatibility, analytical rigor, and societal applicability within a unified sustainability paradigm suitable for modern pharmaceutical analysis.

**Table 6:**
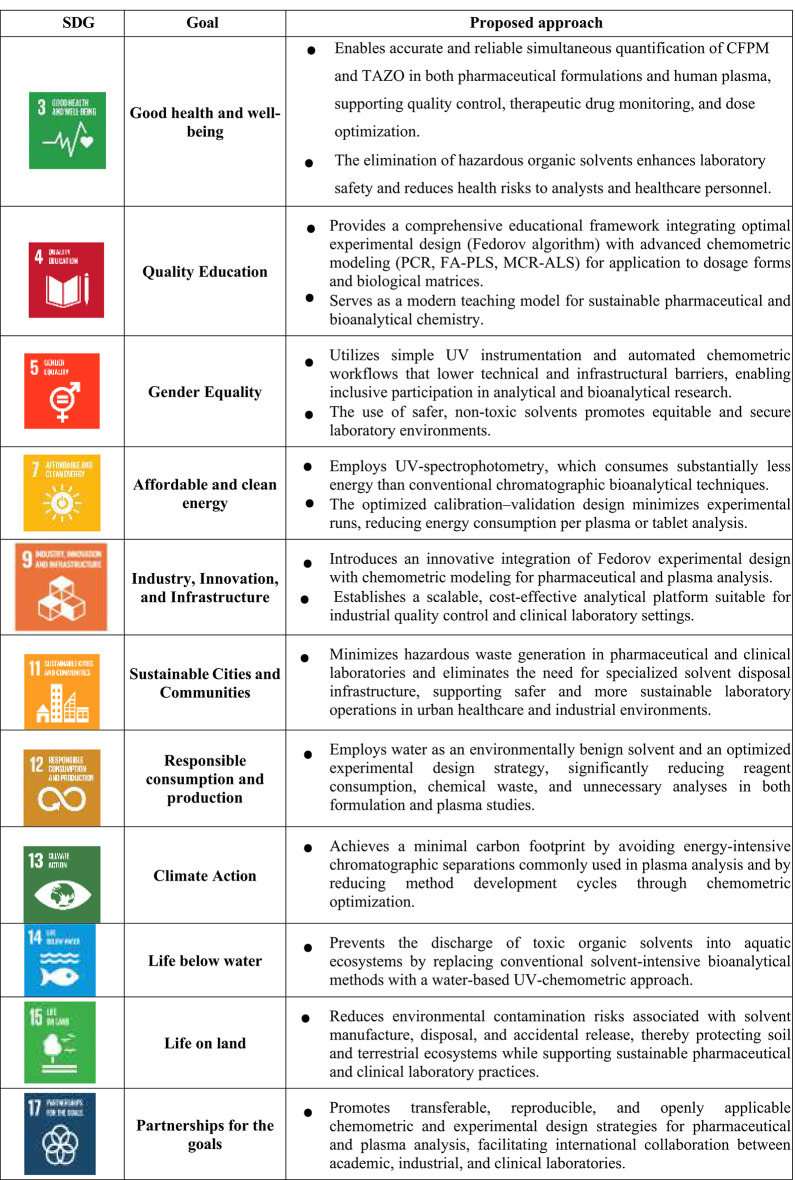
Alignment of the proposed analytical approach with the United Nations Sustainable Development Goals (UN-SDGs 3, 4, 5, 7, 9, 11, 12, 13, 14, 15, and 17).

Additionally, to contextualize the analytical performance of the proposed method, a comparison with representative reported approaches for the simultaneous determination of CFPM and TAZO was conducted. As summarized in Table 7, the proposed MCR-ALS-assisted UV spectrophotometric method offers a well-balanced combination of sensitivity, operational simplicity, minimal solvent consumption, and strong sustainability, while maintaining reliable applicability to both pharmaceutical formulations and plasma samples.

**Table 7:** Comparison of the proposed method with reported analytical methods for cefepime (CEF) and tazobactam (TAZ).

Method	Matrix	Linearity (µg/mL)	LOD (µg/mL)		Sample preparation	Solvent use	Sustainability	Remarks
			CFPM	TAZO				
HPLC-UV^15^	Plasma	5–100 (CFPM) 0.625–12.5 (TAZO)	3.1	0.14	Protein precipitation	Organic solvents	Moderate	Reliable but solvent- intensive
LC-MS/MS^13^	Dog Plasma	0.5–100	0.11	0.09	SPE / PP	High organic use	Low	Very sensitive, high cost
CZE^12^	Plasma	10–100	3.09	1.56	Dilution	Moderate	Moderate	Green alternative but complex, less common
UV Spectrophotometry^18^	Dosage form	5-50 (CFPM) 2.5-17.5 (TAZO)	2.2	1.07	Simple dilution	Minimal	High	Limited selectivity
Proposed method (MCR-ALS)	Plasma & dosage	1–17 (CFPM), 1–9 (TAZO)	0.0487	0.0396	Simple PP	Water only	Excellent	Rapid, High sensitivity, eco-friendly and cost-effective

## Conclusion

This study presents a green, sustainability-oriented UV–spectrophotometric method coupled with advanced chemometric modeling for the simultaneous determination of Cefepime and Tazobactam in pharmaceutical formulations and human plasma. The integration of the Fedorov exchange algorithm for optimal experimental design with PCR, FA-PLS, and MCR-ALS models enabled efficient construction of calibration and validation sets, significantly reducing experimental workload while maintaining statistical rigor and predictive reliability. Comparative evaluation of the chemometric models demonstrated progressive improvement in performance from PCR to FA-PLS and ultimately to MCR-ALS, which consistently provided the highest accuracy, precision, lowest prediction errors, and minimal systematic bias, as evidenced by the smallest Elliptical Joint Confidence Region in both pharmaceutical and biological matrices. The method was successfully validated in accordance with ICH guidelines, demonstrating excellent linearity, sensitivity, precision, and trueness. Reliable quantification was achieved in CEFE-MAX™ powder for injection and fortified human plasma. The successful use of external calibration in plasma analysis, together with negligible matrix effects, confirms strong robustness, calibration transferability, and suitability for rapid screening and preliminary therapeutic monitoring. Comprehensive sustainability assessment highlighted the distinction between greenness and sustainability while confirming the superior environmental profile of the proposed platform. The method achieved a Whiteness Score of 83.6% (MA), an NQS index of 90, and the lowest carbon footprint (0.032 kg CO₂-eq per sample) among the evaluated approaches. Benchmarking against reported HPLC–UV, LC–MS/MS, capillary zone electrophoresis, and conventional UV methods further confirmed a superior balance between analytical performance, environmental impact, and practical applicability. Overall, the proposed strategy demonstrates that the integration of intelligent experimental design with chemometric spectral resolution provides a robust, cost-effective, and environmentally responsible alternative to conventional solvent-intensive methodologies for multicomponent pharmaceutical analysis. The framework offers a scalable approach for aligning analytical performance with modern sustainability principles in future method development.

## Supplementary Information

Below is the link to the electronic supplementary material.

Supplementary Material 1

Supplementary Material 2

## Data Availability

The data used to support the findings of this study are included in the article.
