# Supplementary material for: Fedorov algorithm–optimized chemometric spectrophotometry for cefepime–tazobactam microanalysis in plasma and pharmaceuticals with integrated MA and NQS sustainability assessment
Source: Sci Rep. 2026 Jun 5;16:17526. doi: 10.1038/s41598-026-55675-7 (PMC13241530; doi:10.1038/s41598-026-55675-7)
Supplement: Supplementary file 1 — Supplementary Material 1 [file 41598_2026_55675_MOESM1_ESM.docx]

**Supplementary Figures**


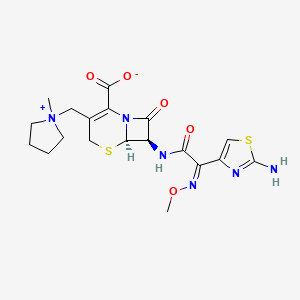


**A**


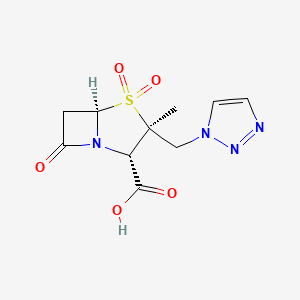


**B**

**Fig. S1**. Chemical structure of (A) CFPM and (B) TAZO.

**Fig. S2.** RMSECV plot of the calibration set as a function of the optimum latent variables (LVs) for the (A) PCR, and (B) FA-PLS models.

**Fig. S3.** Elliptical confidence regions for CFPM and TAZO with the ideal point.


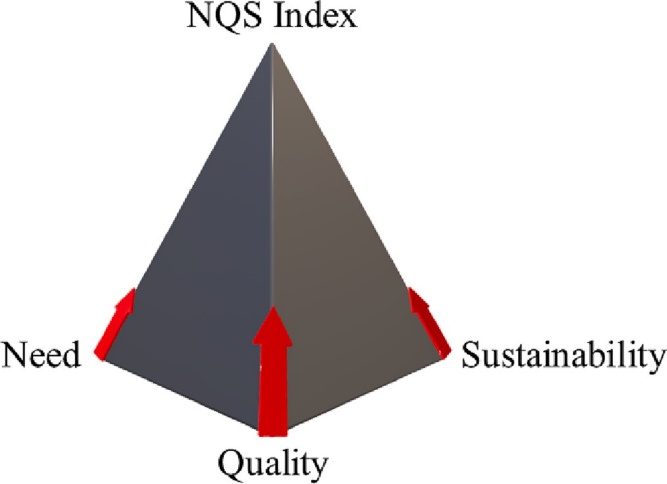


**Fig. S4.** Triangular pyramid illustrating the analytical chemistry for sustainability concept based on the Need – Quality - Sustainability (NQS) index.

**Supplementary Tables**

**Table S1:** Optimized parameters of the Firefly algorithm used for variable selection to enhance the predictability of the PLS models.

| **Parameter** | **CFPM** | **TAZO** |
| --- | --- | --- |
| **Number of fireflies** | **25** | **25** |
| **Maximum number of generations** | **100** | **100** |
| **Randomization parameter (α)** | **0.25** | **0.20** |
| **Attractiveness coefficient (β_ο_)** | **1.0** | **1.0** |
| **Absorption coefficient (γ)** | **0.9** | **0.8** |

**Table S2:** Validation parameters for the determination of CFPM and TAZO in pharmaceutical preparations using the proposed chemometric models.

| **Parameters** | **PCR** | | **GA-PLS** | |
| --- | --- | --- | --- | --- |
|  | **CFPM** | **TAZO** | **CFPM** | **TAZO** |
| **Working range** (μg mL^-1^) | 1–17 | 1–9 | 1–17 | 1–9 |
| **Slope ^a^** | 0.9984 | 0.9976 | 0.9992 | 0.9988 |
| **Intercept ^a^** | 0.0132 | 0.0179 | 0.0086 | 0.0114 |
| **r^2^ ^a^** | 0.9992 | 0.9991 | 0.9996 | 0.9995 |
| **LOD (**μg mL^-1^**) ^b^** | 0.0863 | 0.0724 | 0.0648 | 0.0529 |
| **LOQ (**μg mL^-1^**) ^b^** | 0.2615 | 0.2194 | 0.1964 | 0.1603 |
| **RMSEC ^c^** | 0.326 | 0.301 | 0.248 | 0.221 |
| **RMSEP ^d^** | 0.371 | 0.345 | 0.281 | 0.257 |
| **RRMSEP ^e^** | 2.218 | 2.457 | 1.684 | 1.923 |
| **BCRMSEP ^f^** | 0.129 | 0.118 | 0.082 | 0.074 |
| **SEC ^g^** | 0.341 | 0.315 | 0.262 | 0.236 |
| **Accuracy ^h^ (M.R % ± S.D)** | 99.32 ± 0.618 | 99.56 ± 0.689 | 99.71 ± 0.487 | 99.83 ± 0.532 |
| **Repeatability ^h^ (%RSD )** | 0.653 | 0.724 | 0.492 | 0.548 |
| **Intermediate precision ^h^ (%RSD )** | 0.768 | 0.832 | 0.611 | 0.657 |
| **Robustness ^h^ (%RSD )** | 0.846 | 0.901 | 0.689 | 0.742 |
| **Experimental t value ^i^** | **3.97** | **2.31** | **2.13** | **1.15** |
| **Critical value t_(0.025, 12)_** | **2.179** | **2.179** | **2.179** | **2.179** |
| **Critical value t_(0.01, 12)_** | **2.681** | **2.681** | **2.681** | **2.681** |
|  | | | | |
| **Parameters** | **MCR-ALS** | |  | |
|  | **CFPM** | **TAZO** |  |  |
| **Range (**μg mL^-1^**)** | 1–17 | 1–9 |  |  |
| **Slope ^a^** | 0.9997 | 0.9994 |  |  |
| **Intercept ^a^** | 0.0041 | 0.0063 |  |  |
| **r^2^ ^a^** | 0.9999 | 0.9998 |  |  |
| **LOD** (μg mL^-1^) **^b^** | 0.0487 | 0.0396 |  |  |
| **LOQ** (μg mL^-1^)**^b^** | 0.1476 | 0.1200 |  |  |
| **RMSEC ^c^** | 0.186 | 0.163 |  |  |
| **RMSEP ^d^** | 0.214 | 0.192 |  |  |
| **RRMSEP ^e^** | 1.283 | 1.437 |  |  |
| **BCRMSEP ^f^** | 0.047 | 0.041 |  |  |
| **SEC ^g^** | 0.197 | 0.171 |  |  |
| **Accuracy ^h^ (M.R % ± S.D)** | 100.12 ± 0.352 | 100.05 ± 0.398 |  |  |
| **Repeatability ^h^ (%RSD )** | 0.361 | 0.402 |  |  |
| **Intermediate precision ^h^ (%RSD )** | 0.452 | 0.496 |  |  |
| **Robustness ^h^ (%RSD )** | 0.518 | 0.563 |  |  |
| **Experimental t value ^i^** | **1.22** | **0.45** |  |  |
| **Critical value t_(0.025, 12)_** | **2.179** | **2.179** |  |  |
| **Critical value t_(0.01, 12)_** | **2.681** | **2.681** |  |  |

^a^ Data of the straight line plotted between predicted concentrations versus actual concentrations of the validation set.

^b^ The LOD and LOQ calculations are based on the net analyte signals.

^c^ Root mean square error of calibration.

^d^ Root mean square error of prediction.

^e^ Relative root mean square error of prediction.

^f^ Bias corrected mean square error of prediction.

^g^ standard error of calibration

^h^ Average of three determinations.

^i^t_exp_ = |100 - R̄_exp_| × √n/S_R_, Where: R̄exp is the mean recovery percentage, n is the number of samples (13), and S_R_ is the standard deviation of recoveries.

**Table S3:** One-way ANOVA statistical analysis comparing the performance of three chemometric methods for the simultaneous determination of CFPM and TAZO (95% confidence level)

| **Component** |  | **Sum of squares** | **df** | **Mean square** | **F** | **P value** |
| --- | --- | --- | --- | --- | --- | --- |
| **CFPM** | **Between Groups** | 0.284 | 2 | 0.142 | **1.38** *(3.89)* ^a^ | 0.279 |
|  | **Within Groups** | 2.468 | 24 | 0.103 | — | — |
|  | **Total** | 2.752 | 26 | — | — | — |
|  |  |  |  |  |  |  |
| **TAZO** | **Between Groups** | 0.196 | 2 | 0.098 | **1.11** *(3.89)* ^a^ | 0.346 |
|  | **Within Groups** | 2.119 | 24 | 0.088 | **—** | **—** |
|  | **Total** | 2.315 | 26 | **—** | **—** | **—** |
|  |  |  |  |  |  |  |

^a^ Figures in parentheses represent the corresponding critical value of F at P < 0.05.

**Table S4:** Matrix effect assessment for Cefepime (CFPM) and Tazobactam (TAZO) in human plasma using the optimized MCR-ALS model

| **Analyte** | **Spiked Level (µg mL⁻¹)** | **Calculated Concentration in Neat Solution (µg mL⁻¹) ^a^** | **Calculated Concentration in Post-Extraction Spiked Plasma (µg mL⁻¹) ^a^** | **Response in Neat Solution (a.u.)** | **Response in Post-Extraction Spiked Plasma (a.u.)** | **Matrix Factor (MF) ^b, d^** | **Matrix Effect (%) ^c,e^** |
| --- | --- | --- | --- | --- | --- | --- | --- |
| CFPM | 5.0 | 4.99 | 4.88 | 0.284 | 0.277 | 0.9780 | −2.20 |
|  | 10.0 | 9.97 | 9.81 | 0.566 | 0.555 | 0.9839 | −1.61 |
|  | 15.0 | 14.96 | 14.77 | 0.848 | 0.835 | 0.9873 | −1.27 |
| TAZO | 3.0 | 2.98 | 2.91 | 0.196 | 0.190 | 0.9765 | −2.35 |
|  | 5.0 | 4.99 | 4.88 | 0.327 | 0.319 | 0.9780 | −2.20 |
|  | 7.0 | 6.97 | 6.86 | 0.458 | 0.449 | 0.9842 | −1.58 |

**^a^** Calculated concentrations were obtained using the optimized MCR-ALS chemometric model.
**^b^** Matrix factor (MF) was calculated as the ratio of the calculated concentration in post-extraction spiked plasma to that obtained for the corresponding neat aqueous standard at the same nominal concentration.
**^c^** Matrix effect (%) was calculated as: Matrix Effect (%) = (MF − 1) × 100.
**^d^** Matrix factor values close to unity indicate negligible matrix-induced interference.
**^e^** Negative values indicate signal suppression, positive values indicate signal enhancement, and zero indicates the absence of matrix effect.
