# Supplementary figures and images for: Fedorov algorithm–optimized chemometric spectrophotometry for cefepime–tazobactam microanalysis in plasma and pharmaceuticals with integrated MA and NQS sustainability assessment
Source: Sci Rep. 2026 Jun 5;16:17526. doi: 10.1038/s41598-026-55675-7 (PMC13241530; doi:10.1038/s41598-026-55675-7)

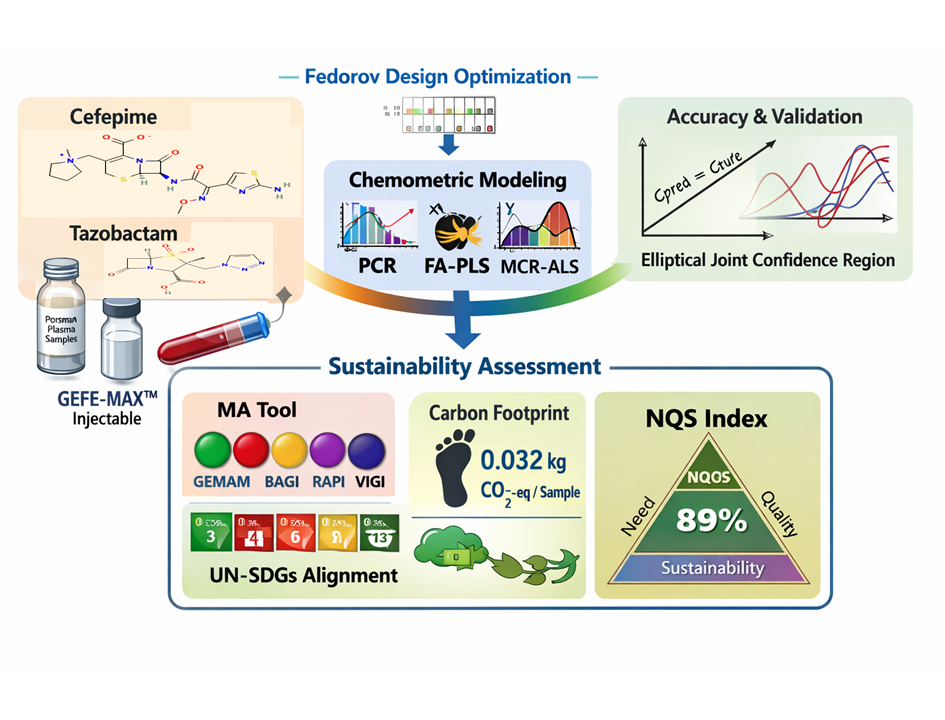

Supplement: Supplementary file 2 — Supplementary Material 2 [file 41598_2026_55675_MOESM2_ESM.tif]
